# Modulation of microRNA expression in human lung cancer cells by the G9a histone methyltransferase inhibitor BIX01294

**DOI:** 10.3892/ol.2014.2034

**Published:** 2014-04-04

**Authors:** ALAN LAP-YIN PANG, ALEXANDRA C. TITLE, OWEN M. RENNERT

**Affiliations:** Laboratory of Clinical and Developmental Genomics, Eunice Kennedy Shriver National Institute of Child Health and Human Development, National Institutes of Health, Bethesda, MD 20892-4429, USA

**Keywords:** G9a methyltransferase, microRNA, lung cancer, H1299, BIX01294

## Abstract

MicroRNAs (miRNAs) are small non-coding RNAs that regulate the expression of their target genes at the post-transcriptional level. In cancer cells, miRNAs, depending on the biological functions of their target genes, may have a tumor-promoting or -suppressing effect. Treatment of cancer cells with inhibitors of DNA methylation and/or histone deacetylation modulates the expression level of miRNAs, which provides evidence for epigenetic regulation of miRNA expression. The consequences of inhibition of histone methyltransferase on miRNA expression, however, have not been thoroughly investigated. The present study examined the expression pattern of miRNAs in the non-small cell lung cancer cell line, H1299 with or without treatment of BIX01294, a potent chemical inhibitor of G9a methyltransferase that catalyzes the mono-and di-methylation of the lysine 9 residue of histone H3. By coupling microarray analysis with quantitative real-time polymerase chain reaction analysis, two miRNAs were identified that showed consistent downregulation following BIX01294 treatment. The results indicate that histone H3 methylation regulates miRNA expression in lung cancer cells, which may provide additional insight for future chemical treatment of lung cancer.

## Introduction

Lung cancer is the leading cause of cancer mortalities worldwide (1). Among all cases, ~80% are classified as non-small cell lung cancer (NSCLC) and the remaining 20% are identified as SCLC. In addition to genetic lesions, including gene mutation, genomic insertion/deletion and translocation, erroneous epigenetic modifications are often involved in the development and progression of cancer (2). Silencing of tumor suppressor genes owing to aberrant promoter DNA methylation (3) and faulty activation of oncogenes caused by genomic DNA hypomethylation (4) are common in cancer cells. Additionally, overexpression of histone deacetylases (HDACs), which induce transcriptional silencing by catalyzing the removal of acetyl moieties from histones, represents another modality of epigenetic defect that contributes to cancer development (5,6). The use of small-molecule chemical agents to reactivate the expression of tumor suppressor genes or to repress oncogenes epigenetically has emerged as a promising approach to eradicate cancer. Accordingly, inhibitors of DNA methyltransferases (DNMTi) and HDACs (HDACi) represent the two major classes of epigenetic antitumor agents.

In addition to protein coding genes, the expression of non-coding RNA transcripts, including microRNAs (miRNAs), is often dysregulated at the epigenetic level in cancer cells (7,8). miRNAs are small RNAs (~22 nucleotides) that regulate gene expression by binding to the 3′-untranslated regions of target gene transcripts to induce translational repression or transcript degradation. Depending on the biological function of the target gene products, miRNAs are involved in diverse biological processes, including cell proliferation and differentiation. With regard to cancer development, miRNAs were shown to exhibit oncogenic (9–11) and tumor suppressive (12–14) properties, respectively. Treatment of cancer cells with HDACi and DNMTi separately or in combination was shown to modulate miRNA expression (15–21), indicating the possibility of suppressing cancer cell growth and spread by targeting miRNA expression.

In addition to DNA methylation and histone acetylation, histone lysine methylation is involved in the epigenetic regulation of gene expression and represents another target of dysregulation. Depending on the position of the lysine residues to be methylated, histone methylation is involved in transcriptional activation and repression. Notably, the mono- and di-methylation of histone H3 at lysine 9 (H3K9me1 and H3K9me2) are associated with transcriptional repression in euchromatin (22). The enzyme responsible for H3K9me1 and H3K9me2 formation is G9a histone methyltransferase (23). G9a expression is upregulated in various types of human cancer (24,25), which indicates that the enzymatic activity is oncogenic. Consistent with this, the promoter regions of the aberrantly silenced tumor suppressor genes are marked by an increased level of H3K9me2 in cancer cells (26), and H3K9me1 and H3K9me2 are erased from the promoters of reactivated tumor suppressor genes (27). Additionally, the silencing of G9a expression by RNA interference reduces the invasiveness and metastatic potential of human lung cancer cells (28) and inhibits the growth of prostate cancer cells (29). These observations indicate a functional association between G9a activity and cancer development. Treatment of cells with BIX01294, a chemical inhibitor specific to G9a, results in a decline of the cellular H3K9me2 content (30). The reduction of proliferation, motility and invasiveness of human neuroblastoma cells following BIX01294 treatment (31) further indicates the use of this chemical as an antitumor agent. To examine whether specific miRNAs are involved in the tumor suppressive effect of G9a inhibition, a microarray analysis was performed in the current study to probe the global change in miRNA expression levels in human NSCLC H1299 cells following BIX01294 treatment.

## Materials and methods

### Cell culture

The human NSCLC cells, H1299 (CRL-5803) were obtained from the American Type Culture Collection (ATCC; Manassas, VA, USA) and cultured in RPMI-1640 medium (Life Technologies, Carlsbad, CA, USA) supplemented with 10% non heat-inactivated fetal bovine serum (ATCC) and 1% antibiotic-antimycotic solution (Corning Inc., Acton, MA, USA). Four hours prior to drug treatment, 5×10^4^ proliferating H1299 cells were seeded into each well of a 12-well culture plate. BIX01294 (Stemgent, Cambridge, MA, USA) was reconstituted in dimethyl sulfoxide (DMSO), and diluted 10 times in 1× phosphate-buffered saline (PBS) immediately prior to use. The working BIX01294 solution was added directly to the culture medium to a final concentration of 4 μM. For the cells that were receiving the mock treatment, an equal volume of PBS-diluted DMSO was added. The cells were incubated at 37°C in a 5% CO_2_ atmosphere for 48 h prior to sample collection.

### Total RNA extraction

H1299 cells were lysed in TRIzol reagent (Life Technologies). The total RNA fraction was harvested following a chloroform extraction and further purified using the Direct-Zol purification kit (Zymo Research Corporation, Irvine, CA, USA). RNA quantity and quality were analyzed using a a NanoVue spectrophotometer (GE Healthcare, Pittsburg, PA, USA) and Bioanalyzer 2100 (Agilent Technologies, Santa Clara, CA, USA), respectively.

### miRNA microarray analysis

A genome-wide miRNA expression profiling experiment was performed by LC Sciences (Houston, TX, USA). The probes for a total of 2,019 unique mature human miRNAs (Sanger miRBase Release 19.0; Wellcome Trust Sanger Institute, Hinxton, UK) were printed on the microarray in quadruplicate. Equal quantities of total RNA from three independent preparations of each sample group (mock versus BIX01294 treatment) were pooled for the miRNA microarray experiment. Fluorescent signals were background subtracted and normalized using the locally weighted scatterplot smoothing method. A two-tailed t-test (P<0.01 was identified to indicate a statistically significant difference) was performed to identify the differentially expressed miRNAs.

### Quantitative real-time polymerase chain reaction (qPCR) analysis of miRNAs

qPCR analysis of the expression level of individual miRNAs was performed according to instructions from Life Technologies (User bulletin no. 4465407, Jan 2013 version C) with minor modifications. Briefly, reverse transcription (RT) was conducted using the Taqman microRNA Reverse Transcription kit. In each reaction, 50 ng total RNA was reverse transcribed in the presence of 6 μl 100-fold diluted RT primer stock solution, 2 mM deoxyribonucleotide triphosphate, 3.8 units of RNase inhibitor and 150 units of MultiScribe reverse transcriptase. The RT product was diluted five times in nuclease-free water. In each subsequent PCR reaction, 8 μl of the diluted RT product was used with 1× Taqman microRNA assay and 1× Taqman Universal Master Mix (Life Technologies). Triplicate measurements for each miRNA were performed, and the analysis was performed with the three independent preparations of total RNA samples harvested from each sample group. The expression level of individual miRNAs was normalized to that of small nucleolar RNA, RNU24, and the change in expression level was calculated using the 2^−ΔΔCt^ method. A two-tailed t-test (P<0.05 was identified to indicate a statistically significant difference) was performed to identify the differentially expressed miRNAs. All Taqman miRNA assays for mature human miRNAs and RNU24 were purchased from Life Technologies.

### miRNA target prediction

Potential target genes of miRNAs were predicted using the miRNA Target Prediction and Functional Study Database (www.mirdb.org) (32,33). Enrichment analysis for disease-associated genes was performed using the WEB-based Gene Set Analysis Toolkit (http://bioinfo.vanderbilt.edu/webgestalt/) (34). The functional annotation of the genes was further inquired from the Gene References Into Functions (GeneRIFs) on the National Center for Biotechnology Information website (http://www.ncbi.nlm.nih.gov/gene/).

### Statistical analysis

All statistical analyses were performed using a two-tailed Student’s t-test. P<0.01 and P<0.05 were considered to indicate a statistically significant difference in miRNA microarray and qPCR analysis, respectively.

## Results

### G9a regulates the expression of miRNAs in human lung cancer cells

To examine whether G9a regulates the expression of miRNAs in human lung cancer cells, a microarray analysis was performed to study the change in the global miRNA expression pattern in H1299 cells in the presence and absence of BIX01294. Among the 2,019 mature human miRNAs scrutinized, only 51 of them were found to be differentially expressed (Table I). To identify the miRNAs that showed a robust change in expression level, the focus was on the miRNAs that exhibited a signal intensity of 500 units in at least one of the sample groups (35). Of the 51 differentially expressed miRNAs, 10 passed this selection criterion. Among the 10 miRNAs, six showed downregulation and the remaining four showed upregulation, following BIX01294 treatment.

**Figure 1:**
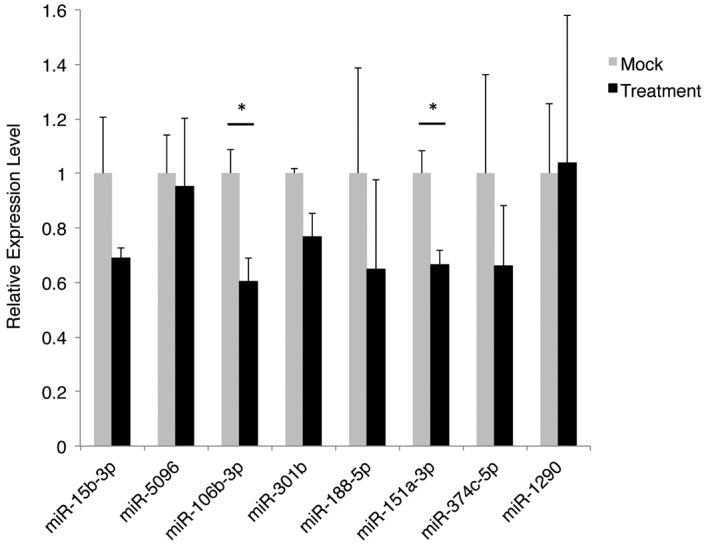
Quantitative polymerase chain reaction analysis of selected miRNAs between H1299 cells receiving mock and BIX01294 treatment. Each bar represents the average expression level of the corresponding miRNA from a triplicate measurement of three independent preparations of total RNA samples. ^*^P<0.05. miRNA, microRNA.

Subsequently, a qPCR experiment was performed to validate the differential expression patterns of the 10 miRNAs. At the time of the experiment, the Taqman miRNA assay for one of the miRNAs (hsa-miR-1229-5p) was not available; therefore, it was excluded from the analysis. In addition, hsa-miR-3613-3p was found to be undetectable in the sample groups in qPCR analysis. The normalized and averaged expression levels of the remaining eight miRNAs are shown in Fig. 1. Two of the miRNAs, hsa-miR-106b-3p and hsa-miR-151a-3p, exhibited a significant reduction (40 and 33%, respectively) in expression level that is consistent with the result that was obtained from the microarray analysis.

### Certain target genes of hsa-miR-151a-3p are associated with cancerous diseases

A search was conducted for the genes whose expression may be regulated by these two miRNAs. A total of 14 and 182 genes were predicted to be the targets of hsa-miR-106b-3p and hsa-miR-151a-3p, respectively (Table II). The small number of genes identified for hsa-miR-106b-3p precluded the performance of a robust prediction of the associated biological functions or disorders. A gene ontology analysis of the 182 genes that were potentially regulated by hsa-miR-151a-3p revealed that the most significantly associated biological process was the negative regulation of branching that is involved in ureteric bud morphogenesis. To examine if there is an association of human diseases with the predicted target genes, an enrichment analysis for disease-associated genes was performed. A total of 10 classes of disease (neoplasm metastasis, neoplastic processes, syndrome, adhesion, carcinoma, fasciculation, eye abnormalities, brain injuries, schizophrenia, optic nerve diseases) were found to be associated with the 182 genes. Among these diseases neoplasm metastasis, neoplastic processes, adhesion and carcinoma are relevant to cancer development and propagation. The genes associated with these four diseases are listed in Table III.

## Discussion

The involvement in cell growth and susceptibility to epigenetic dysregulation highlights the role of miRNAs in cancer development. For this reason, these small RNA species may serve as potential therapeutic targets against cancer. The role of H3K9 methylation in the regulation of miRNA expression in human lung cancer cells has not been established. In the present study, it was found that the blockade of G9a activity, and thus histone H3K9 methylation, modulated the expression of miRNAs in the invasive H1299 lung cancer cell line. By interrogating the change in miRNA expression pattern with microarray analysis, it was found that only a particularly small portion of the human miRNA collection (51 out of 2,019; 2.5%) exhibited differential expression following BIX01294 treatment. This observation indicates that the regulatory activity of G9a may be specific towards a subset of miRNAs in these cells. Coupled with qPCR analysis, the two miRNAs that were identified, hsa-miR-106b-3p and hsa-miR-151a-3p, were downregulated in H1299 cells following BIX01294 treatment.

The biological function of hsa-miR-106b-3p and hsa-miR-151a-3p in lung cancer development has not been characterized. Based on their genomic location, hsa-miR-106b-3p and hsa-miR-151a-3p are known to reside in chromosome 7q22.1 and 8q24.3, respectively. The two genomic loci are frequently amplified in various cancers and the overexpression of the embedded genes has been shown to promote malignancy (36–40). Furthermore, the amplified and overexpressed functional non-coding RNA species participate in cancer development (41). Therefore, the downregulation of hsa-miR-106b-3p and hsa-miR-151a-3p expression by BIX01294 treatment may exert a tumor suppressive effect, presumably through the derepression of their target gene expression at the post-transcriptional level. The silencing of G9a expression inhibits the migration and invasion potential of lung cancer cells by enhancing the transactivation of expression of the cell adhesion molecule, epithelial cell adhesion molecule (28). It is likely that specific target genes of these miRNAs may encode adhesion molecules, which promote cell-cell adhesion and limit cell motility.

The target genes of hsa-miR-106b-3p and hsa-miR-151a-3p were searched for, and their biological activities and associated human diseases were identified. The small number of genes identified for hsa-miR-106b-3p precluded the performance of a robust prediction of their associated biological functions or human diseases. For hsa-miR-151a-3p, it was found that a subset of its target genes is involved in the cell adhesion process. Notably, certain genes (PCDHB7, PTPN12, CHL1, PPF1A1 and THBS1) encode cell adhesion or cell junction molecules that have been demonstrated or indicated to inhibit cell invasion (42–45). In addition, the genes exhibiting a similar function (TFAP2C, PCDHB13 and MNT) (46,47) are also target genes of hsa-miR-106b-3p. As a result, the inhibition of G9a activity by BIX01294 treatment may suppress metastasis by downregulating the expression level of miRNAs that block the translation of genes encoding the cell adhesion molecules. By contrast, another subset of the target genes of hsa-miR-151a-3p were identified, which are involved in neoplasm formation and metastasis; however, their involvement in the action of BIX01294 on H1299 cells remains unclear. The extent of derepression of the individual target genes may determine the overall cellular response to the downregulation of hsa-miR-106b-3p and hsa-miR-151a-3p. Alternatively, the biological effect of BIX01294 on H1299 cells may involve the interplay among these gene products.

The change in expression level of specific miRNAs upon inhibition of G9a activity strongly indicates a role for miRNAs in the mediation of the malignancy-promoting effect of G9a. Since mono- and di-methylation of H3K9 are involved in transcriptional silencing (22), a blockade of G9a activity, and thus H3K9me1 and H3K9me2 formation, is expected to reactivate the transcription of genes, including miRNAs. By contrast, the mechanism of downregulation of miRNA expression by G9a suppression remains unclear and requires further investigation.

In conclusion, the findings of the present study indicate that the suppression of G9a activity by BIX01294 treatment modulates the expression of specific miRNAs in H1299 cells. Further studies are required to establish the functional role, and prognostic and diagnostic potential of hsa-miR-106b-3p and hsa-miR-151a-3p in lung cancer development.

## Figures and Tables

**Table I:** Human miRNAs that showed a differential expression in H1299 cells following BIX01294 treatment through microarray analysis.

Reporter term	Mock		Treatment		Fold change (Treatment/Mock)	P-value	miRNA sequence (5′ to 3′)
	
	Mean	SD	Mean	SD			
Transcripts with high microarray signal levels (signal >500)							
hsa-miR-15b-3p	604	24	336	20	0.56	1.72×10^−5^	CGAAUCAUUAUUUGCUGCUCUA
hsa-miR-5096	3,782	492	1,289	318	0.34	1.40×10^−3^	GUUUCACCAUGUUGGUCAGGC
hsa-miR-106b-3p	545	19	311	33	0.57	2.39×10^−3^	CCGCACUGUGGGUACUUGCUGC
hsa-miR-1229-5p	260	29	576	137	2.22	4.12×10^−3^	GUGGGUAGGGUUUGGGGGAGAGCG
hsa-miR-301b	982	50	433	94	0.44	5.11×10^−3^	CAGUGCAAUGAUAUUGUCAAAGC
hsa-miR-188-5p	675	93	1,128	192	1.67	5.33×10^−3^	CAUCCCUUGCAUGGUGGAGGG
hsa-miR-151a-3p	665	61	364	73	0.55	5.96×10^−3^	CUAGACUGAAGCUCCUUGAGG
hsa-miR-374c-5p	1,257	48	747	112	0.59	7.68×10^−3^	AUAAUACAACCUGCUAAGUGCU
hsa-miR-3613-3p	2,960	195	9,363	3,142	3.16	8.55×10^−3^	ACAAAAAAAAAAGCCCAACCCUUC
hsa-miR-1290	1,205	73	2,211	390	1.83	9.56×10^−3^	UGGAUUUUUGGAUCAGGGA
Transcripts with low microarray signal levels (signal <500)							
hsa-miR-335-5p	49	13	0	0	0.00	1.08×10^−5^	UCAAGAGCAAUAACGAAAAAUGU
hsa-miR-1207-5p	231	12	343	24	1.49	2.86×10^−4^	UGGCAGGGAGGCUGGGAGGGG
hsa-miR-550a-3-5p	100	6	157	13	1.57	3.07×10^−4^	AGUGCCUGAGGGAGUAAGAG
hsa-miR-577	44	27	0	0	0.00	5.14×10^−4^	UAGAUAAAAUAUUGGUACCUG
hsa-miR-4440	200	13	147	9	0.74	8.02×10^−4^	UGUCGUGGGGCUUGCUGGCUUG
hsa-miR-1303	108	19	50	7	0.46	1.16×10^−3^	UUUAGAGACGGGGUCUUGCUCU
hsa-miR-5707	253	36	447	43	1.76	1.16×10^−3^	ACGUUUGAAUGCUGUACAAGGC
hsa-miR-501-5p	157	21	89	12	0.57	1.48×10^−3^	AAUCCUUUGUCCCUGGGUGAGA
hsa-miR-16-2-3p	393	23	239	28	0.61	1.69×10^−3^	CCAAUAUUACUGUGCUGCUUUA
hsa-miR-657	64	9	34	5	0.53	1.71×10^−3^	GGCAGGUUCUCACCCUCUCUAGG
hsa-miR-4669	254	25	394	42	1.55	1.90×10^−3^	UGUGUCCGGGAAGUGGAGGAGG
hsa-miR-224-5p	368	28	203	33	0.55	2.14×10^−3^	CAAGUCACUAGUGGUUCCGUU
hsa-miR-548au-5p	25	19	0	0	0.00	2.15×10^−3^	AAAAGUAAUUGCGGUUUUUGC
hsa-let-7a-3p	120	27	51	11	0.43	2.22×10^−3^	CUAUACAAUCUACUGUCUUUC
hsa-miR-4749-3p	166	12	113	13	0.68	2.34×10^−3^	CGCCCCUCCUGCCCCCACAG
hsa-miR-6511b-3p	241	48	117	9	0.49	2.45×10^−3^	CCUCACCACCCCUUCUGCCUGCA
hsa-miR-339-5p	233	25	154	7	0.66	2.46×10^−3^	UCCCUGUCCUCCAGGAGCUCACG
hsa-miR-596	154	10	112	11	0.73	2.56×10^−3^	AAGCCUGCCCGGCUCCUCGGG
hsa-miR-101-3p	339	13	185	23	0.55	2.76×10^−3^	UACAGUACUGUGAUAACUGAA
hsa-miR-4258	201	17	134	16	0.67	2.99×10^−3^	CCCCGCCACCGCCUUGG
hsa-miR-339-3p	115	15	71	10	0.61	3.48×10^−3^	UGAGCGCCUCGACGACAGAGCCG
hsa-miR-3156-5p	168	12	273	41	1.62	3.80×10^−3^	AAAGAUCUGGAAGUGGGAGACA
hsa-miR-130b-5p	228	11	137	16	0.60	3.83×10^−3^	ACUCUUUCCCUGUUGCACUAC
hsa-miR-4417	98	9	132	10	1.36	4.41×10^−3^	GGUGGGCUUCCCGGAGGG
hsa-miR-6073	112	30	318	94	2.84	4.64×10^−3^	GGUAGUGAGUUAUCAGCUAC
hsa-miR-3943	94	10	33	9	0.35	4.78×10^−3^	UAGCCCCCAGGCUUCACUUGGCG
hsa-miR-371a-5p	98	9	143	18	1.45	5.20×10^−3^	ACUCAAACUGUGGGGGCACU
hsa-miR-30d-3p	177	12	70	18	0.40	5.32×10^−3^	CUUUCAGUCAGAUGUUUGCUGC
hsa-miR-493-3p	71	13	116	14	1.63	5.85×10^−3^	UGAAGGUCUACUGUGUGCCAGG
hsa-miR-140-3p	283	9	200	19	0.71	5.96×10^−3^	UACCACAGGGUAGAACCACGG
hsa-miR-4278	124	30	307	88	2.47	6.25×10^−3^	CUAGGGGGUUUGCCCUUG
hsa-miR-539-3p	60	23	171	45	2.86	6.39×10^−3^	AUCAUACAAGGACAAUUUCUUU
hsa-miR-654-5p	57	20	19	7	0.34	7.03×10^−3^	UGGUGGGCCGCAGAACAUGUGC
hsa-miR-24-2-5p	291	24	192	28	0.66	7.33×10^−3^	UGCCUACUGAGCUGAAACACAG
hsa-miR-140-5p	86	31	27	8	0.32	7.62×10^−3^	CAGUGGUUUUACCCUAUGGUAG
hsa-miR-3162-3p	127	6	108	6	0.85	7.76×10^−3^	UCCCUACCCCUCCACUCCCCA
hsa-miR-296-5p	222	28	159	13	0.72	8.19×10^−3^	AGGGCCCCCCCUCAAUCCUGU
hsa-miR-222-5p	237	29	91	27	0.38	8.24×10^−3^	CUCAGUAGCCAGUGUAGAUCCU
hsa-miR-564	285	19	193	30	0.68	8.65×10^−3^	AGGCACGGUGUCAGCAGGC
hsa-miR-642b-3p	241	24	333	40	1.38	8.93×10^−3^	AGACACAUUUGGAGAGGGACCC
hsa-miR-576-5p	86	29	25	13	0.29	8.96×10^−3^	AUUCUAAUUUCUCCACGUCUUU

SD, standard deviation.

**Table II:** Putative target genes of hsa-miR-106b-3p and hsa-miR-151a-3p.

miRNA	Putative target gene
hsa-miR-106b-3p	LOC347411, SOCS7, TFAP2C, C15orf26, PCDHB13, C4orf39, RNGTT, ARHGAP17, IRAK2, TNRC6A, BICD2, KCTD2, SLC35A1, MNT
hsa-miR-151a-3p	UPP2, FXR1, PKN2, ZFAND5, GABRA6, ZMAT1, KCNH8, ME1, CLASP2, ZNF326, PGM3, RPS6KA5, EIF2C2, ATP2A2, CLK1, PITPNA, CHL1, GFM2, DCTN4, ITK, HIF1A, CASD1, FAM104A, LIG4, SIX1, FAM76B, ADAM7, PURB, RGS6, CRK, KLHL4, AQP4, SLC8A1, ANKRD44, PTGER3, HMGN2, OXR1, TRA2B, API5, FAM5C, STXBP4, ZFPM2, RYBP, YSK4, ZNF24, ACTR2, PTPRZ1, ARMC8, NEURL1B, PANK2, PANX3, CAPZA2, ARHGAP23, ZEB1, DISC1, SNX18, MFAP5, FAM59A, TRDN, RERG, FBXL3, QKI, GHR, CCNDBP1, ZNF415, C5orf28, DBT, TSC1, CREBZF, ZNF254, IL26, SPIRE2, PFN2, RBM27, CEP95, RNF20, SOS1, RBM5, DSCC1, CAB39, NKAIN3, PCBP2, CYTIP, LPIN2, C1orf9, PTPN12, MANEA, SOCS5, DTX3L, YTHDF3, SLCO3A1, TWIST1, PRKACB, CUX1, MRPS25, PYROXD1, IKZF3, HSDL1, BTLA, PLEKHF2, CAST, YIPF6, SETD6, C9orf117, GPD2, C3orf43, CGA, CASC4, HMGA2, SPIRE1, DTHD1, OSBPL3, ACAP2, GLS, LOC100506156, SERPINA1, PHC1, FAM199X, SUSD5, RAB3GAP1, KIAA1217, PCDHB7, C15orf41, ECT2L, SIAH3, OPA3, STMN2, FAM211A, LGSN, SLA, NIPBL, SEC22C, BMPR1B, HCCS, ZSCAN29, TAF5L, LOC100653121, PPFIA1, DUSP19, HPDL, UCHL1, HTR1F, SLC35E2B, ZNF345, GREM1, TET2, NIPAL2, LOC100652774, C3orf17, FAM170A, MYLK4, CEACAM5, ARL1, NETO2, SLC35E2, THBS1, C8orf84, SMARCAD1, PGR, TMEM99, KBTBD2, CALD1, DSG3, GRM3, PGM2L1, LAPTM5, UHMK1, TACSTD2, MAGEA2B, FAM120AOS, PAPOLG, C10orf10, ZNF264, MAGEA2, PRR23C, CALCR, PTPRS, RPRD2, TIAM1, NAMPT, MCTP2, RPL27A

**Table III:** Putative target genes of hsa-miR-151a-3p that are associated with diseases relevant to cancer.

Human disease	Putative target gene
Neoplasm metastasis	SIX1, HIF1A, ZEB1, TACSTD2, TWIST1, CEACAM5, TIAM1, PGR, THBS1
Neoplastic processes	HMGA2, SIX1, CRK, HIF1A, ZEB1, TWIST1, CEACAM5, TIAM1, PGR, THBS1
Adhesion	CYTIP, PCDHB7, CRK, PTPN12, TACSTD2, CEACAM5, CHL1, PPFIA1, TIAM1, DSG3, THBS1
Carcinoma	RBM5, HMGA2, SIX1, HIF1A, ZEB1, TWIST1, CEACAM5, TIAM1, PGR, THBS1
